# Supplementary material for: Bandgap Engineering of Two-Dimensional Double Perovskite Cs4AgBiBr8/WSe2 Heterostructure from Indirect Bandgap to Direct Bandgap by Introducing Se Vacancy
Source: Materials (Basel). 2023 May 11;16(10):3668. doi: 10.3390/ma16103668 (PMC10221128; doi:10.3390/ma16103668)
Supplement: Supplementary file 1 [file materials-16-03668-s001.zip › materials-2317657-supplementary.pdf]

Supplementary Material

Figure S1 shows the optimized geometry of $Cs_4AgBiBr_8/WSe_2$ heterostructure with defect. The structural deformation of the heterojunction is larger with W vacancy than with Se vacancy.

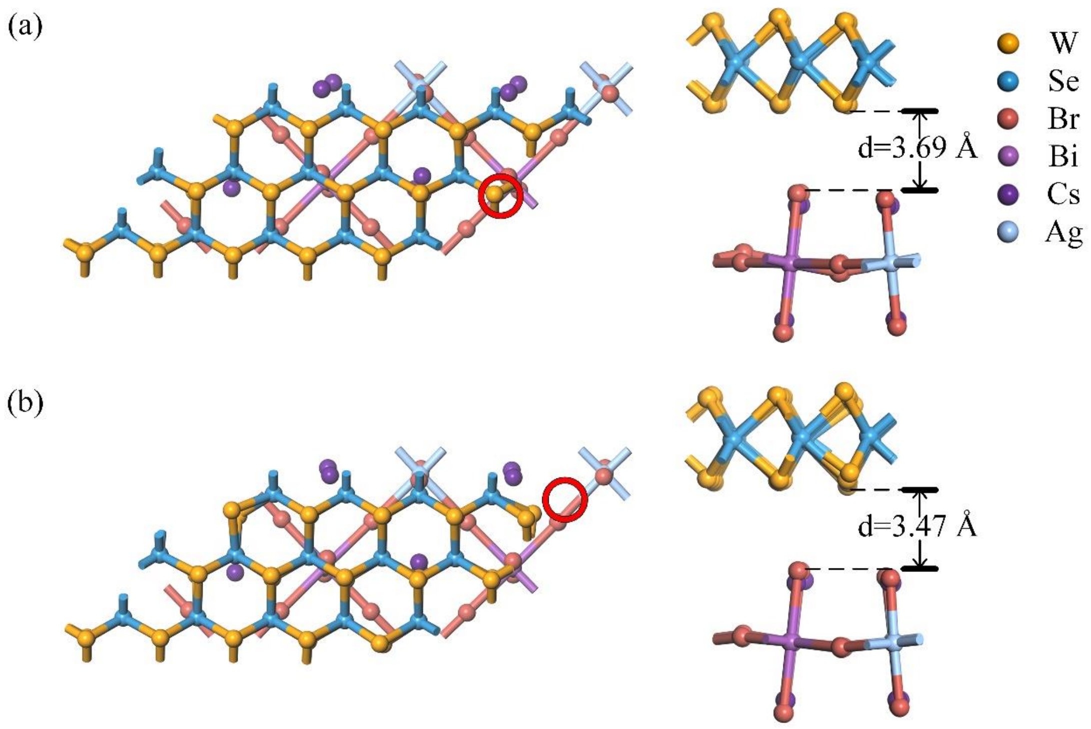

Figure S1. The top and side views of $Cs_4AgBiBr_8/WSe_2$ heterostructure with (a) Se vacancy and (b) W vacancy. The positions of the vacancies are marked with red circles.

Figure S2 shows the band structures and DOS of those heterostructures which have Se vacancy. When Se vacancy appears at different positions in the heterostructure, their electronic properties are similar.

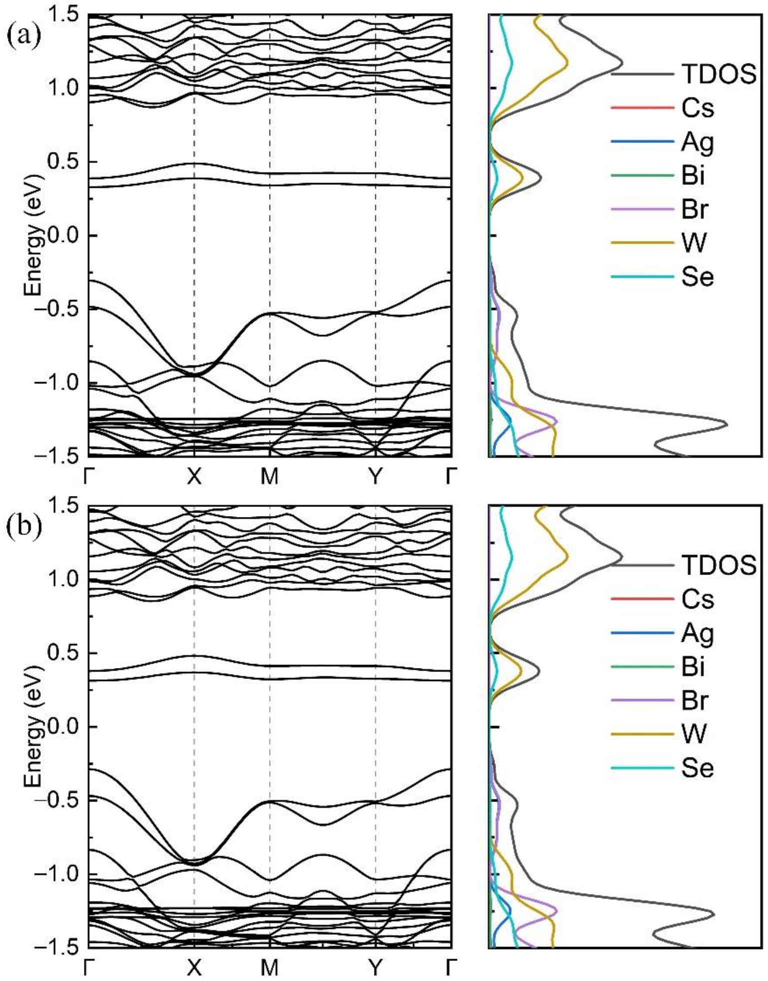

Figure S2. The band structures (left) and the DOS (right) of the $Cs_4AgBiBr_8/WSe_2$ heterostructure with (a) No. 2 Se vacancy and (b) No. 3 Se vacancy.

Table S1 shows the adhesion energy of $Cs_4AgBiBr_8/WSe_2$ heterostructures with different Se atom vacancy defects, which are calculated according to the following equations:

$$E_{ad} = E_{hete.} - E_{WSe_2} - E_{Cs_4AgBiBr_8},$$

and

$$E_{WSe_2} + E_{Cs_4AgBiBr_8} = -401.2952\ eV,$$

where $E_{hete.}$, E_{WSe_2}, and $E_{Cs_4AgBiBr_8}$ represent the total energies of the relaxed heterostructures $WSe_2/Cs_4AgBiBr_8$, pure monolayer WSe_2, and pure monolayer $Cs_4AgBiBr_8$, respectively.

Table S1. The adhesion energy (eV) of 30 $Cs_4AgBiBr_8/WSe_2$ heterostructures with different Se atom vacancy defects.

**No.**	**1**	**2**	**3**	**4**	**5**	**6**
$E_{hete.}$	—401.5181	–401.5199	–401.5182	–401.5190	–401.5160	–401.5435
E_{ad}	–0.2229	–0.2246	–0.2229	–0.2238	–0.2208	–0.2482
No.	7	8	9	10	11	12
$E_{hete.}$	–401.5141	–401.5208	–401.5179	–401.5267	–401.5160	–401.5221
E_{ad}	–0.2188	–0.2256	–0.2227	–0.2314	–0.2208	–0.2269
No.	13	14	15	16	17	18

$E_{hete.}$	–401.5144	–401.5424	–401.5199	–401.5219	–401.5167	–401.5107
E_{ad}	–0.2191	–0.2472	–0.2247	–0.2267	–0.2214	–0.2155
No.	19	20	21	22	23	24
$E_{hete.}$	–401.5202	–401.5219	–401.5188	–401.5207	–401.5173	–401.5205
E_{ad}	–0.2250	–0.2266	–0.2236	–0.2254	–0.2221	–0.2252
No.	25	26	27	28	29	30
$E_{hete.}$	–401.5129	–401.5155	–401.5173	–401.5225	–401.5170	–401.5226
E_{ad}	–0.2177	–0.2202	–0.2221	–0.2272	–0.2217	–0.2274
